# Structural alterations and markers of endothelial activation in pulmonary and bronchial arteries in fatal asthma

**DOI:** 10.1186/s13223-019-0363-0

**Published:** 2019-08-28

**Authors:** Renata Calciolari Rossi, Raquel Anonni, Diogenes Seraphim Ferreira, Luiz Fernando Ferraz da Silva, Thais Mauad

**Affiliations:** 1 Department of Pathology, Universidade de São Paulo-School of Medicine, Avenida Dr. Arnaldo, 455., CEP 01246-903 São Paulo, SP Brazil; 2 Department of Pathology, Universidade do Oeste Paulista, Presidente Prudente, São Paulo Brazil; 3 Department of Physiotherapy, Universidade Federal do Triângulo Mineiro, Uberaba, Minas Gerais Brazil; 4 Allergy and Immunology, Hospital das Clínicas, Federal University of Paraná, Curitiba, Brazil

**Keywords:** Asthma, Pulmonary artery, Bronchial artery, Pathology, Extracellular matrix, Adhesion molecules, Endothelial activation, Remodelling

## Abstract

**Background:**

There is interest in better understanding vessel pathology in asthma, given the findings of loss of peripheral vasculature associated with disease severity by imaging and altered markers of endothelial activation. To date, vascular changes in asthma have been described mainly at the submucosal capillary level of the bronchial microcirculation, with sparse information available on the pathology of bronchial and pulmonary arteries. The aim of this study was to describe structural and endothelial activation markers in bronchial arteries (BAs) and pulmonary arteries (PAs) of asthma patients who died during a fatal asthma attack.

**Methods:**

Autopsy lung tissue was obtained from 21 smoking and non-smoking patients who died of an asthma attack and nine non-smoking control patients. Verhoeff–Masson trichrome staining was used to analyse the structure of arteries. Using immuno-histochemistry and image analyses, we quantified extracellular matrix (ECM) components (collagen I, collagen III, versican, tenascin, fibronectin, elastic fibres), adhesion molecules [vascular cell adhesion molecule 1 (VCAM-1) and intercellular adhesion molecule 1 (ICAM-1)] and markers of vascular tone/dysfunction [endothelin-1 (ET-1) and angiotensin II type 2 receptor (AT2)] in PAs and BAs.

**Results:**

There were no significant differences in ECM components, ICAM-1, ET-1 or AT2 between asthma patients and controls. Smoking asthma patients presented with decreased content of collagen III in both BA (p = 0.046) and PA (p = 0.010) walls compared to non-smoking asthma patients. Asthma patients had increased VCAM-1 content in the BA wall (p = 0.026) but not in the PA wall.

**Conclusion:**

Our data suggest that the mechanisms linking asthma and arterial functional abnormalities might involve systemic rather than local mediators. Loss of collagen III in the PA was observed in smoking asthma patients, and this was compatible with the degradative environment induced by cigarette smoking. Our data also reinforce the idea that the mechanisms of leukocyte efflux via adhesion molecules differ between bronchial and pulmonary circulation, which might be relevant to understanding and treating the distal lung in asthma.

## Background

Asthma is a chronic inflammatory disease associated with structural changes in the lungs, mainly at the airway level [1]. Vascular changes in asthma have been mostly described at the submucosal capillary level of the bronchial microcirculation and include angiogenesis, vascular dilation and hyperpermeability. These features are thought to contribute to the maintenance of inflammation and airway thickening [2].

It has to be emphasized that the arterial supply of the lungs occurs via two different systems. The bronchial arteries (BAs) arise from the aorta or intercostal arteries and supply blood to the airways, tracheobronchial lymph nodes, and nerves. The pulmonary artery (PA) runs within the airway connective tissue sheath, branching with the airways. It supplies the distal lung and is considered the functional artery of the lung [3]. Little is known about human pathology of the pulmonary and bronchial arteries in asthma.

In animals, house dust mite (HDM)-induced allergic inflammation caused pulmonary vascular remodelling that did not revert entirely after allergen withdrawal [4]. A study using the Severe Asthma Research Program Cohort detected pruning of the pulmonary vasculature in asthma by computed tomography that was associated with lower lung function, higher eosinophilia and an increased rate of exacerbation [5]. However, few studies have addressed the structure of the pulmonary and bronchial lung arteries in asthma. Green et al. described thickening of the intimal layer associated with narrowing of the lumen of BAs, suggesting that it could contribute to bronchial hyperresponsiveness [6]. On the other hand, Saetta et al. did not detect thickening of the peripheral PA in six patients who died of acute onset asthma [7], despite the presence of eosinophilic/mast cell inflammation [8]. No studies have addressed the composition of the extracellular matrix (ECM) that forms the artery walls in human asthma.

Additionally, there are differences in leukocyte trafficking between the bronchial (airways) and pulmonary circulation (distal lung), possibly due to the type and size of vessels, function and expression of adhesion molecules and differences in stimuli that induce inflammatory responses at these sites [9]. A large epidemiological study has shown that asthmatic patients have an increased risk of developing (local or systemic) pulmonary thromboembolism, with higher risk in asthma patients with more exacerbations and hospitalizations [10]. Signs of endothelial activation in PAs [11] and an increase in coagulation factors in severe asthma have been previously described in asthma and could account for this epidemiological finding [12]. There is no information related to the tissue expression of adhesion molecules comparing bronchial and pulmonary circulation in asthma.

Cigarette smoking is associated with several adverse effects in asthma [13]. Cigarette smoking is also known to cause alterations in pulmonary vessel structure and endothelial cell dysfunction [14], but the extent to which vascular the alterations present in smoking asthma patients contribute to asthma severity is unknown. Endothelin-1 (ET-1), a potent vasoconstrictor, is involved in a pathway of tobacco-induced vascular dysfunction in smokers [15], and it is considered a mediator of asthma [16]. Angiotensin II type 2 receptor (AT2) has been described as protective against vasoconstriction, proliferation and fibrosis in models of acute lung injury and bronchodysplasia [17, 18], but there is no information about the expression of AT2 in the lungs of asthma patients.

To address these questions, we analysed structural ECM components (collagen I, collagen III, versican, tenascin, and fibronectin), adhesion molecules [vascular cell adhesion molecule 1 (VCAM-1) and Intercellular Adhesion Molecule 1 (ICAM 1)] and markers of vascular tone/dysfunction (ET-1 and AT2) in pulmonary and bronchial arteries of smoking and non-smoking patients who died due to an acute asthma attack. We hypothesized that bronchial and pulmonary arteries have differential expression of these markers and that the differences would be more pronounced in smoking asthma patients.

## Methods

### Study population

This study was approved by the human studies review board of the São Paulo University Medical School (CAPPesq-HCMUSP). Tissue was obtained from lung of 21 patients who died due to asthma attack (fatal asthma). All subjects had a previous history of asthma, documented by interviews with the families, and had no other lung disease. All patients were autopsied at the Department of Pathology of the São Paulo University between 2004 and 2006. All patients had their deaths ascribed to asthma by a pathologist. Of the 21 patients, 10 were smokers and 11 were non-smokers. Pathological inclusion criteria were lung hyperinflation and mucus hypersecretion and the following histological changes related to asthma: epithelial shedding, basement membrane thickening, airway smooth muscle hypertrophy and cellular inflammatory infiltrate with or without eosinophils. Nine non-smoking subjects who died of non-pulmonary causes and had no previous history of asthma or any other pulmonary disease were included as controls. A mean of 14 h elapsed between death and autopsy for both asthmatics and controls. This material has been used in previous studies [19, 20].

### Tissue processing for immunohistochemistry

Random samples from central and peripheral areas of the lung were collected from all patients. Tissue was fixed in 10% buffered formalin, routinely processed and embedded in paraffin. Sections 5-µm thick were cut and stained with H&E, modified Verhoeff–Masson trichrome (structural analysis) and oxidized resorcin-fuchsin (ORF, elastic fibres) [21].

In addition, sections were deparaffinized, and endogenous peroxidase was blocked with a 0.5% hydrogen peroxide in methanol solution for 10 min at room temperature. Pre-treatment of slides for antigen retrieval was performed with citrate for fibronectin, VCAM-1, ET-1, AT2 and collagen I and III staining, with trypsin for versican staining, with TRIS–EDTA for ICAM-1 staining and with pepsin for collagen III and tenascin. Sections were incubated overnight in a humid chamber with primary antibodies (Table 1). The streptavidin–biotin complex LSAB (Dako, Glostrup, Denmark) was used after secondary antibodies. Negative controls were performed by replacing primary antibody with phosphate-buffered saline and by substituting primary antibody with an isotype-matched antibody control.

**Table 1:** Antibodies used for immunohistochemical analyses

Antibody	Pre-treatment	Dilution autopsy tissue	Clone	Manufacturer
Collagen I	Citrate	1:2500	Polyclonal	US Biological, Swampscott, MA, USA
Collagen III	Pepsin	1:15		Abcam, Cambridge, USA
Fibronectin	Citrate	1:12000	Polyclonal	Dako, Glostrup, Denmark
Tenascin	Pepsin	1:20000	BC-24	Sigma-Aldrich, Missouri, USA
Versican	Trypsin	1:1000	2-b-1	Seikagaku Co. Tokyo, Japan
AT2	Citrate	1:1000	EPR3876	Abcam, Cambridge, USA
Endothelin-1	Citrate	1:300		Santa Cruz Biotechnology, CA, USA
ICAM-1	Tris–EDTA	1:50	23G12	Abcam, Cambridge, USA
VCAM-1	Citrate	1:100		Santa Cruz Biotechnology

*AT2* angiotensin II type 2 receptor, *ET-1* endothelin-1, *ICAM-1* intercellular adhesion molecule 1, *VCAM-1* vascular cell adhesion molecule 1

### Image analysis and quantification

Five PAs adjacent to peripheral airways that were cut into transverse sections were selected for each case. We also selected five BAs cut in transverse sections present in the outer large airway walls (external to the airway smooth muscle layer). For both artery types, the vessel diameter was determined as the shortest diameter between two points of the external elastic lamina in a plane perpendicular to the long axis of each artery [22]. All slides were scanned using a 3DHistec device (3DHistec, Budapest, Hungary), and image analysis was performed with Image Pro-Plus 4.1 software for Windows (Media Cybernetics Silver Spring, MD, USA), installed on a personal computer [8]. Using modified Verhoeff-Masson trichrome staining, we assessed the following structural parameters: lumen area and artery wall area (intima and muscle layers). The content of elastic fibres was assessed with ORF staining. All values were normalized to the perimeter of the external smooth muscle layer.

The expression of VCAM-1, ICAM-1, ET-1, AT2, fibronectin, versican, tenascin, collagen I and III in the PA and BA walls was calculated as integrated optical density (IOD), the product of mean colour density (MCD) (range 0–255) and positive areas (µm^2^). We divided IOD per positive area to obtain the isolated mean density from 0 to 255, with 0 representing black and 255 representing white. To combine the area and colour, we obtained the inverse results of mean density (MD) using 255—MCD (so that the more intensely stained areas would have a greater value) multiplied by the positive area. The expression of the measured markers was normalized to the total area and expressed as MD* µm^2^/µm^2^. This index of protein expression accounts for both the colour intensity and the stained area. Measurements were performed by a blinded observer for all groups (RCR) [23].

### Statistical analysis

Variables are presented as the mean ± SD or median (IQR) according to data distribution determined by the Kolmogorov-Smirnoff test. T test or Mann–Whitney tests were used to compare groups (asthma vs. control, smoking vs. non-smoking asthma patients). The correlations were analysed by the Spearman or Pearson tests, depending on the distribution of the sample. Values p < 0.05 were considered statistically significant. Analyses were performed using the SPSS 15.0 statistical package for Windows (SPSS Inc., Chicago, IL, USA).

## Results

The subject’s characteristics are shown in Table 2. The median (range) age of the asthma patients and controls was 44 (17–66) and 52 (44–63) years, respectively (p < 0.001). Only 25% of the asthma patients were regularly followed by a doctor; 33% had been hospitalized due to an asthma exacerbation in the previous year, and 12% had had a previous intensive care unit admission due to asthma. Smoking asthma patients had a median of 20 (13–60) pack-years of smoking. There were no significant differences in the demographic characteristics of non-smoking fatal asthma and smoking fatal asthma subjects (data not shown).

**Table 2:** Characteristics of fatal asthma patients and control subjects

	Fatal asthma	Controls
Subjects n (M/F)	21 (12/9)	9 (5/4)
Age (years)	44 (17–66)	52 (44–63)
Non-smoking/smoking (n)	11/10	9/0
Disease onset (years)	17 (1–59)	–
Duration of disease (years)	26 (5–62)	–
Corticosteroid, systemic or inhaled n (%)	12 (57)	0
Systemic corticosteroid n (%)	9 (75)	0
Inhaled corticosteroid n (%)	1 (8)	0
Inhaled and systemic corticosteroid n (%)	2 (17)	0
Short-acting bronchodilator n (%)	21 (100)	0
Cause of death (n)		
Asthma	21	
Cardiovascular disease		8
Acute pancreatitis		1

The morphological characteristics of PAs and BAs are shown in Tables 3 and 4. The diameter of the analysed arteries was not different between asthma patients and controls. There were no differences in arterial wall and lumen areas when asthma patients (smoking and non-smoking subjects) and controls were compared for both PA and BA.

**Table 3:** Structural characteristics of pulmonary and bronchial arteries in asthma patients and controls

Structure	Bronchial artery			Pulmonary artery		
	Asthma	Control	p	Asthma	Control	p
Diameter (µm)	328.14 ± 168.77	436.66 ± 237.71	0.16	480.42 ± 373.37	285.11 ± 156.54	0.14
Wall area^a^	21.17 ± 5.99	25.25 ± 13.99	0.42	23.01 ± 7.63	17.39 ± 5.25	0.055
Lumen area^a^	26.55 ± 12.57	33.02 ± 16.90	0.25	43.42 ± 24.74	31.64 ± 10.39	0.076
Elastic fibres^a^	2.18 ± 2.53	2.14 ± 1.89	0.969	7.11 ± 7.41	4.98 ± 3.76	0.424

Data are presented as the mean ± SD

^a^Wall, lumen areas and elastic fibre stained areas were normalized to the outer smooth muscle layer perimeter and are expressed as µm^2^/µm

**Table 4:** Structural characteristics of pulmonary and bronchial arteries in smoking and non-smoking asthma patients

Structure	Bronchial artery			Pulmonary artery		
	Smoking asthma patients	Non-smoking asthma patients	p	Smoking asthma patients	Non-smoking asthma patients	p
Diameter (µm)	334.40 ± 192.08	322.45 ± 153.92	0.876	388.20 ± 285.78	564.27 ± 192.08	0.292
Wall area^a^	22.01 ± 4.88	20.4 ± 7.01	0.55	22.68 ± 8.97	23.31 ± 6.63	0.86
Lumen area^a^	24.66 ± 13.32	28.27 ± 12.22	0.52	40.25 ± 20.94	46.30 ± 28.47	0.59
Elastic fibres^a^	1.87 ± 1.36	2.45 ± 3.31	0.613	4.97 ± 3.05	9.05 ± 9.62	0.215

Data are presented as the mean ± SD

^a^The wall, lumen and elastic fibres stained areas were normalized to the outer smooth muscle layer perimeter and are expressed as µm^2^/µm

**Fig. 1:**
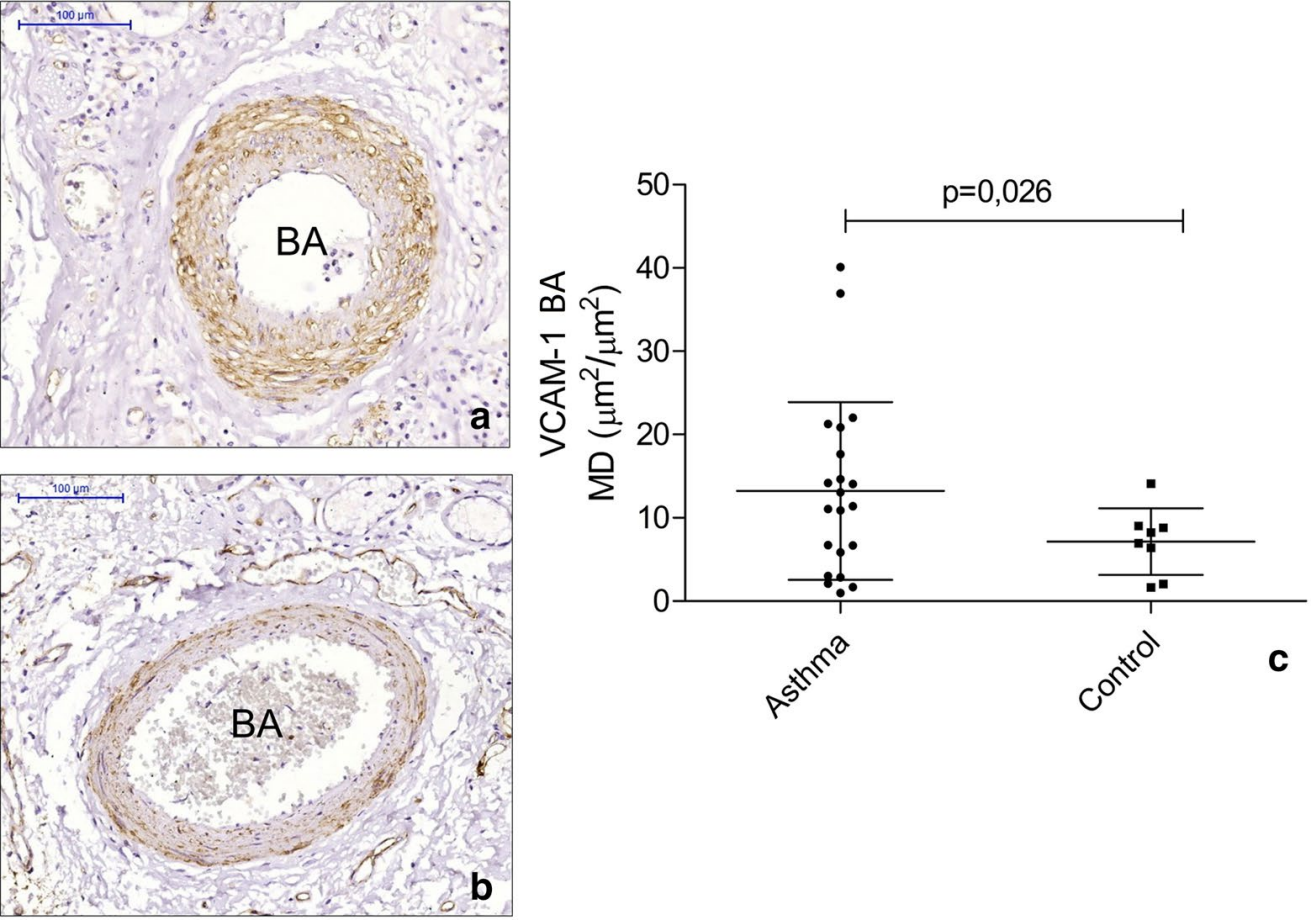
Figure depicts the expression of vascular cell adhesion molecule 1 (VCAM-1) in bronchial arteries of asthma patients (**a**) and controls (**b**), with increased expression in **a**. In **c** the lines represent the mean and standard deviation

**Fig. 2:**
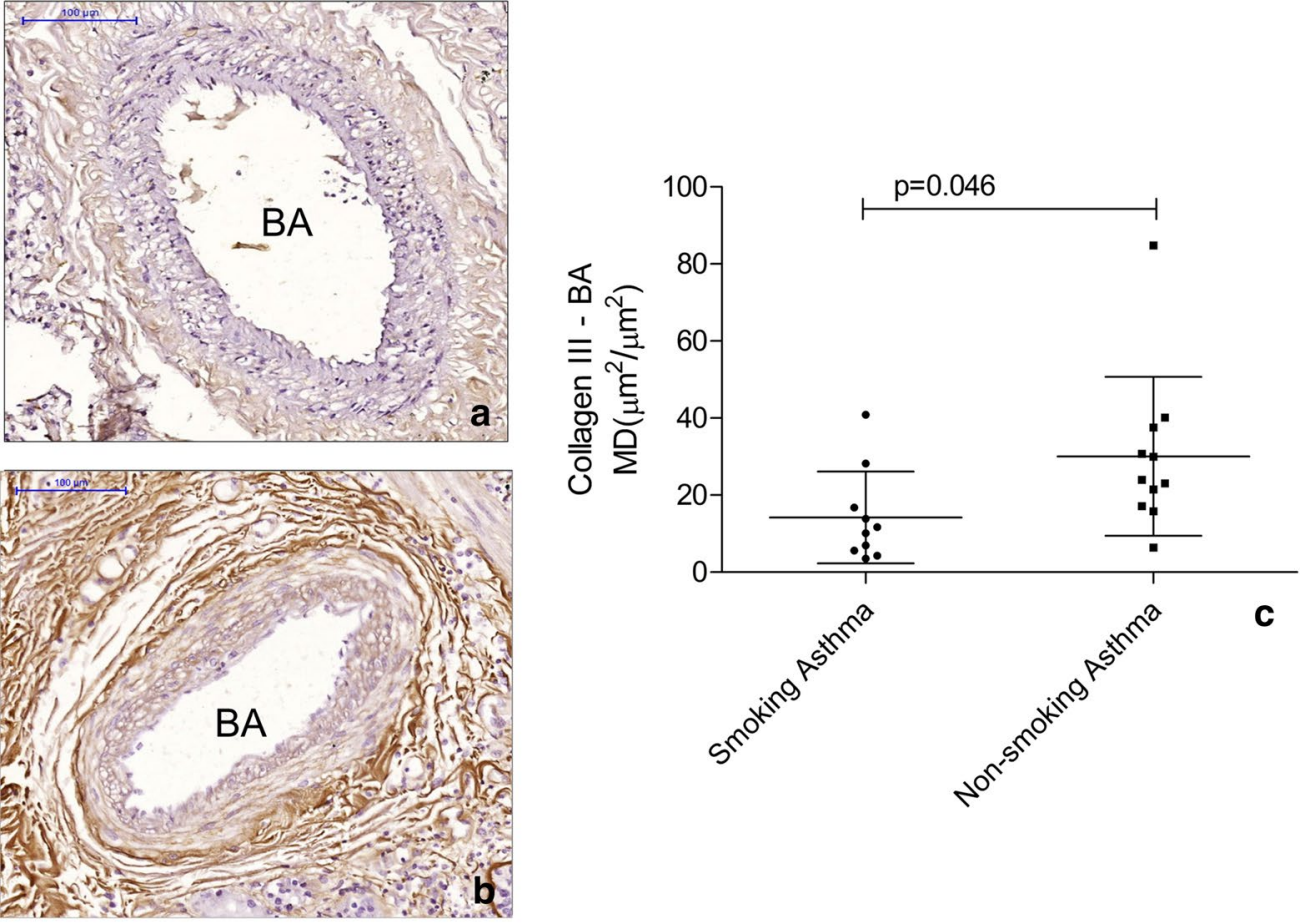
Figure depicts the decreased expression of collagen III in bronchial arteries (BA) of smoking asthma subjects (**a**) when compared to non-smoking asthma subjects (**b**). In **c** the lines represent the mean and standard deviation

**Fig. 3:**
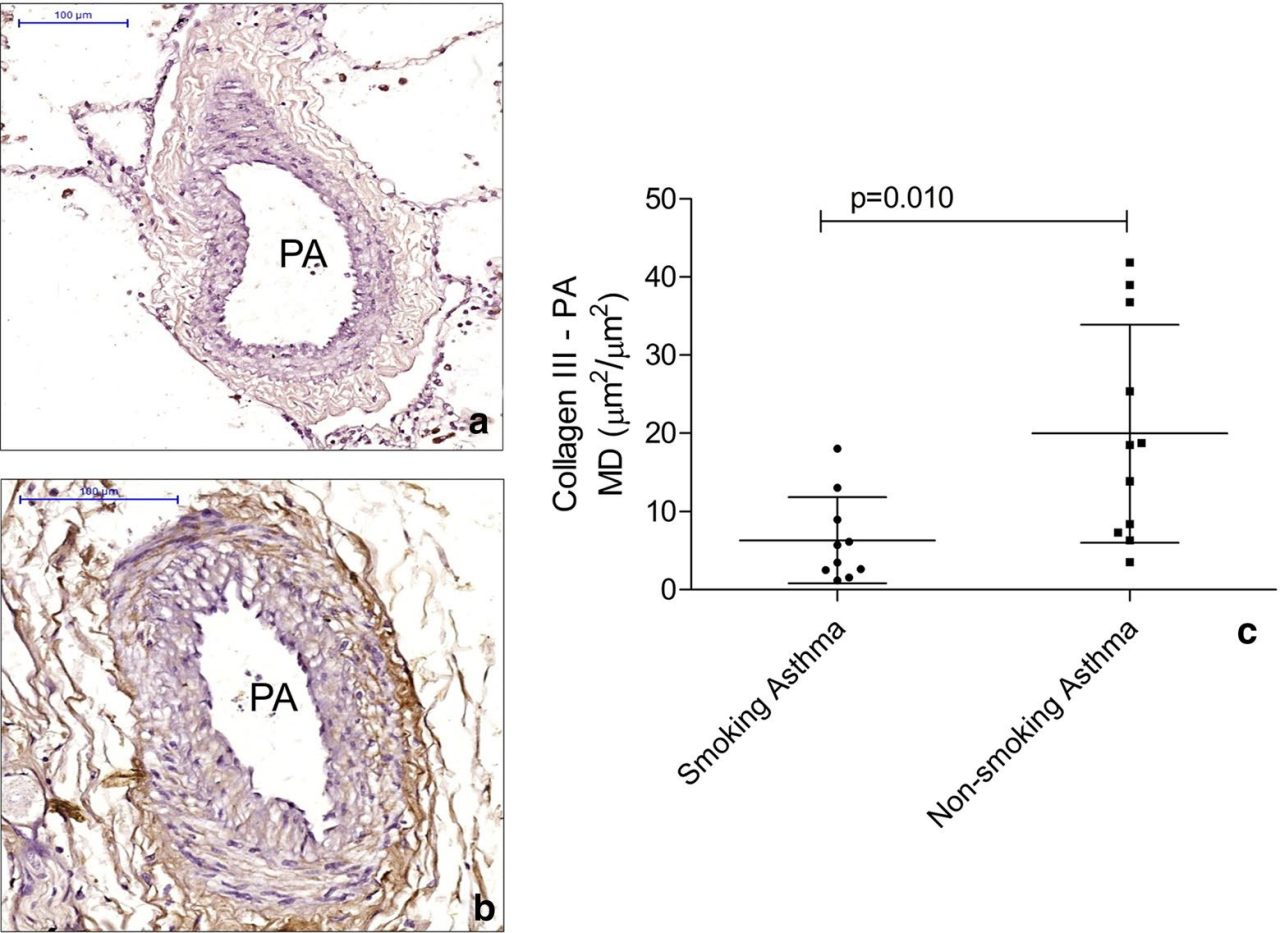
Figure depicts the decreased expression of collagen III in pulmonary arteries (PA) of smoking asthma patients (**a**) when compared to non-smoking asthma patients (**b**). In **c** the lines represent the mean and standard deviation

**Fig. 4:**
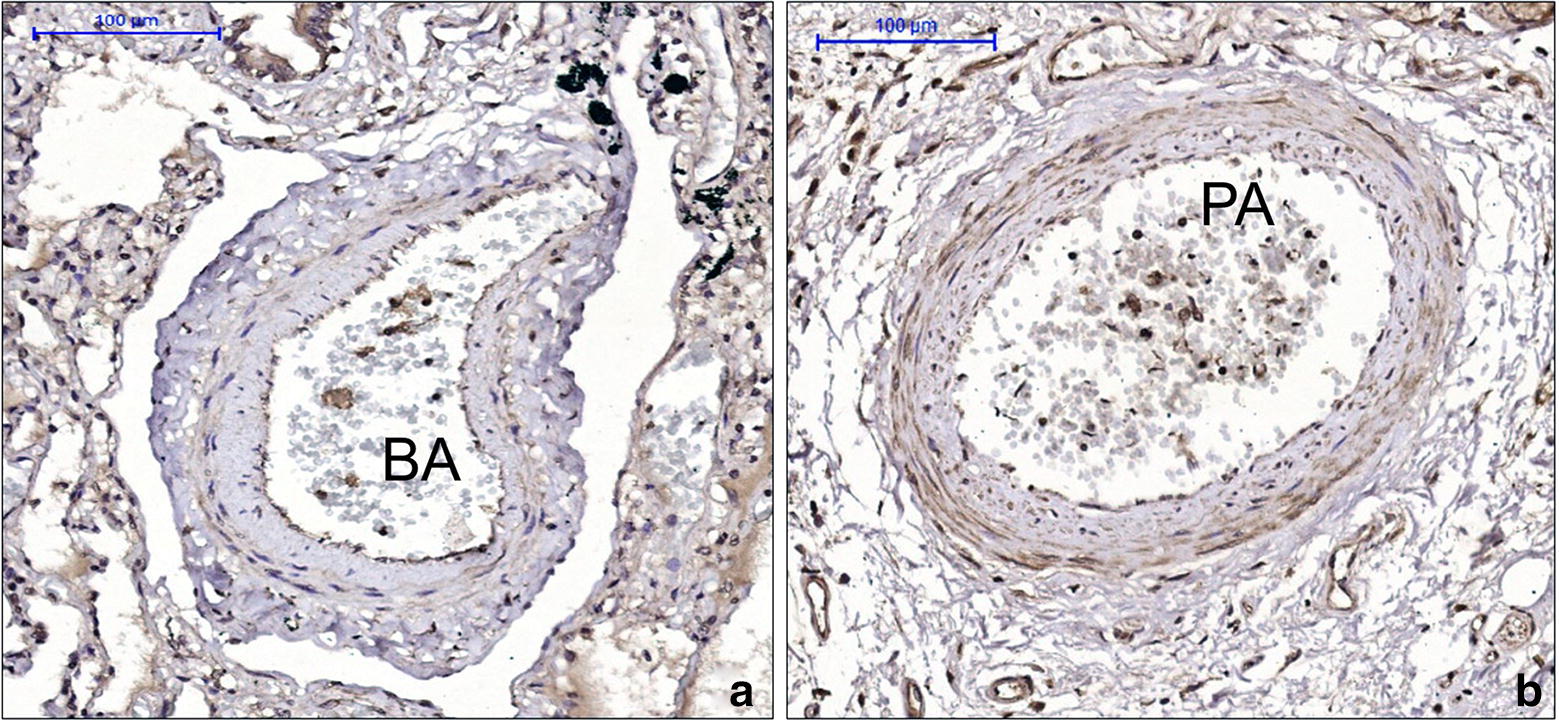
Positive staining of angiotensin II type 2 receptor (AT2) was observed in the walls of the bronchial artery (BA) and pulmonary artery (PA), which did not have differential expression between control and asthma patients

There were no differences between asthma patients and controls for collagen I, collagen III, tenascin, fibronectin, versican and elastic fibre content in both PAs and BAs (Table 5). Asthma patients had increased VCAM-1 content in the BA wall (p = 0.026) but not in PAs (Fig. 1). Smoking asthma patients presented with decreased content of collagen III in both BA (p = 0.046) (Fig. 2) and PA (p = 0.010) (Fig. 3) walls compared to non-smoking asthma patients. There were no differences in the expression of ICAM-1, AT2 (Fig. 4) and ET-1 in both types of arteries for (smoking and non-smoking) asthma patients and controls (Tables 5 and 6).

**Table 5:** Comparison between the expression of extracellular matrix components and endothelial activation markers in pulmonary and bronchial arteries in asthma patients and controls

Marker	Bronchial artery			Pulmonary artery		
	Asthma	Control	p	Asthma	Control	p
Collagen I	0.59 (0.21–2.58)^a^	0.33 (0.08–4.69)^a^	0.689	5.15 ± 0.96	9.10 ± 3.79	0.340
Collagen III	22.51 ± 4.03	17.77 ± 6.30	0.527	17.16 (8.52–30.31)^a^	10.88 (3.25–32.29)^a^	0.533
Tenascin	38.39 ± 5.53	49.73 ± 11.23	0.315	30.92 (17.24–60.90)^a^	41.99 (20.41–81.83)^a^	0.449
Fibronectin	23.26 ± 3.02	32.52 ± 11.23	0.289	47.43 ± 4.70	45.18 ± 8.19	0.803
Versican	8.96 ± 2.24	7.44 ± 2.37	0.692	26.12 ± 3.16	16.96 ± 4.23	0.113
AT-2	7.84 ± 1.56	16.14 ± 6.32	0.235	8.29 ± 1.09	11.89 ± 2.95	0.287
ET-1	31.53 ± 6.19	36.19 ± 7.94	0.670	21.40 ± 2.77	26.27 ± 6.75	0.519
ICAM-1	5.10 ± 1.06	4.41 ± 1.84	0.735	5.56 ± 0.67	4.94 ± 0.88	0.603
VCAM-1	13.19 ± 10.71	3.97 ± 7.28	0.026	9.80 ± 6.20	9.72 ± 8.49	0.980

Data represent the mean density

*AT-2* angiotensin II type 2 receptor, *ET-1*: endothelin-1, *ICAM-1* intercellular adhesion molecule 1

^a^Data are presented as the mean ± SD or median (IQR)

**Table 6:** Comparison between the expression of extracellular matrix components and endothelial activation markers in pulmonary and bronchial arteries in smoking and non-smoking asthma patients

Marker	Bronchial artery			Pulmonary artery		
	Smoking asthma patients	Non-smoking asthma patients	p	Smoking asthma patients	Non-smoking asthma patients	p
Collagen I	1.82 ± 1.47	1.13 ± 2.21	0.412	5.38 ± 5.03	4.95 ± 4.05	0.83
Collagen III	14.20 ± 11.92	30.08 ± 20.60	0.046	6.33 ± 5.53	19.96 ± 13.94	0.010
Tenascin	44.93 ± 25.08	41.08 ± 28.46	0.743	2.87 (0.67–5.31)^a^	1.01 (0.51–7.94)^a^	0.60
Fibronectin	25.95 ± 17.55	27.91 ± 31.61	0,865	54.53 ± 25.71	46.97 ± 20.95	0.462
Versican	9.39 ± 11.71	7.91 ± 8.16	0.739	20.03 ± 15.12	28.37 ± 12.36	0.182
AT-2	9.98 ± 5.37	5.99 ± 8.33	0.224	6.75 ± 2.13	9.70 ± 6.44	0.177
ET-1	43.82 ± 32.35	20.05 ± 19.90	0.054	24.78 ± 12.93	18.33 ± 12.31	0.256
ICAM-1	6.83 ± 5.97	3.53 ± 3.14	0.142	6.65 ± 3.70	4.58 ± 2.15	0.144
VCAM-1	15.12 ± 10.96	11.42 ± 10.77	0.443	9.94 ± 7.63	9.67 ± 4.93	0.92

Data represent the mean density

^a^Data are presented as the mean ± SD or median (IQR)

*AT-2* angiotensin II type 2 receptor, *ET-1* endothelin-1, *ICAM-1* intercellular adhesion molecule 1, *VCAM-1* vascular cell adhesion molecule 1

There were no significant correlations of VCAM-1 and collagen III expression with age, pack-years, disease onset and duration of asthma in asthma patients or controls (data not shown).

## Discussion

In this study, we analysed the structure, ECM composition and markers of endothelial activation in pulmonary and bronchial arteries of patients who died due to a fatal asthma attack. There were no significant differences in structural alterations or ECM composition between asthma patients and controls in both pulmonary and bronchial arteries. Smoking asthma patients had a decreased content of collagen III in both types of artery walls. VCAM-1 expression was increased only in the BA walls, suggesting that differences exist in endothelial activation between pulmonary and bronchial arteries in asthma. To our knowledge, this is the first study addressing the structure and markers of endothelial activation in pulmonary and bronchial arteries in asthma.

There has been a recent renewed interest in better understanding vessel pathology in asthma. Ash et al. described vascular pruning in severe asthma that was related to several parameters of disease severity [5], and previous animal studies suggested the presence of remodelling in the PAs in models of allergic sensitization [24]. We could not find differences in structure or ECM composition in pulmonary or bronchial arteries in asthma. Our data confirm previous data by Saetta et al. in distal pulmonary arteries of 6 cases of fatal asthma [7]. On the other hand, Green et al. found an increased thickness of the intimal layer in bronchial artery branches in asthma that was related to the duration of disease in asthma [6]. The reasons for the discrepant data are not clear and are possibly related to patient characteristics in both cohorts, such as the use of corticosteroids. Our data suggest that the mechanisms leading to vessel pruning are probably functional rather than anatomical but do not exclude the influence of perivascular inflammation on the control of vascular tone [8].

Smoking is associated with endothelial dysfunction and pulmonary vessel remodelling [14]. In this study, we found that patients with asthma who smoked had less collagen III in both artery types. In patients with chronic obstructive pulmonary disease, markers of collagen III degradation are related to overall mortality and clinical outcomes [25]. It would also be interesting to investigate whether lung collagen degradation in smoker contributes to increased severity of asthma.

Previous evidence indicated that asthma causes inflammation of pulmonary arteries, as measured by PA rings of asthma patients who underwent surgery [11]. We have previously described an increase in eosinophils, mast cells and neutrophils in the adventitial region of distal pulmonary arteries [8]. However, in this study, VCAM-1 expression was increased only in the BA walls of asthma patients but not in the PAs, and there were no significant differences in endothelin-1 and ICAM-1 expression. VCAM-1 is an adhesion molecule that is important for regulating the arrest and recruitment of circulating eosinophils during allergic inflammation [26, 27]. The mechanisms regulating leukocyte kinetics between pulmonary and bronchial circulation are likely to be different. These include differences in the diameter of the vessels, differences in the cell types and adhesion molecules that regulate the response, and differences in the stimuli that induce the inflammatory response at these sites [9]. Our data support the idea that leukocyte efflux in the microinflammatory milieu of airways occurs differently than in the distal lung in asthma, with the involvement of different adhesion molecules. Leukocytes come in closer contact with the endothelium for prolonged periods under normal conditions in smaller pulmonary vessels and capillaries, so the cascade occurring in systemic vessels may not be relevant for pulmonary capillaries [28].

In patients with acute severe asthma, the renin-angiotensin system is activated [29]. Angiotensin II has been implicated in the muscularization of pulmonary vessels during hypoxia [30, 31]. The expression of the AT2 receptor had not been studied in human lungs with asthma thus far. More specifically, in contrast to the AT1 receptor, the AT2 receptor is believed to have antifibrotic and antiproliferative effects [32]. The beneficial role of AT2 receptor could be associated with the absence of structural changes in arteries in asthma. We detected expression of the AT2 receptor in artery walls, but no differential expression was observed in asthma patients compared to that in controls.

This study has several limitations. We did not have full clinical and functional characterization of these patients to perform significant correlations, since most of these patients had no regular treatment or were inadequately treated [33]. Due to the retrospective nature of this study, we were not able to perform stereological sampling of lungs, which would have enabled us to perform vessel volume analysis. Nevertheless, autopsy material is thus far the most appropriate way to understand larger vessel pathology, rather than bronchial capillary pathology, in asthma.

## Conclusion

No structural changes or differences in ECM compositions were observed in pulmonary or bronchial arteries of patients who died of asthma in comparison to those in controls. Taken together, our data suggest that vascular pathology in asthma is influenced by functional/systemic factors rather than local factors. Loss of collagen III in both types of arteries was observed in smoking asthma patients, which was compatible with the degradative environment induced by cigarette smoking. Our data also reinforce the idea that mechanisms of leukocyte efflux via adhesion molecules differ between bronchial and pulmonary circulation, which might be relevant to understanding and treating the distal lung in asthma.

## Data Availability

All of the available data are presented in the manuscript. For access to the research material, contact tmauad@usp.br.

## Abbreviations

AT1: angiotensin II type 1 receptor
AT2: angiotensin II type 2 receptor
BA: bronchial artery
ECM: extracellular matrix
ET-1: endothelin-1
H&E: hematoxylin and eosin
HDM: house dust mite
ICAM-1: intercellular adhesion molecule 1
ICU: intensive care unit
IOD: integrated optical density
IQR: interquartile range
LSAB: streptavidin–biotin complex
M/F: male/female
MCD: mean colour density
MD: mean density
µm^2^: square micrometres
p: p value
PA: pulmonary artery
RFO: oxidized resorcin fuchsin
SD: standard deviation
TRIS–EDTA: hydroxymethyl aminomethane–ethylenediamine tetra-acetic acid
VCAM-1: vascular cell adhesion molecule 1
