# Correction to: Structural alterations and markers of endothelial activation in pulmonary and bronchial arteries in fatal asthma

**DOI:** 10.1186/s13223-020-00490-6

**Published:** 2020-10-21

**Authors:** Renata Calciolari Rossi, Raquel Annoni, Diogenes Seraphim Ferreira, Luiz Fernando Ferraz da Silva, Thais Mauad

**Affiliations:** 1 Department of Pathology, Universidade de São Paulo-School of Medicine, Avenida Dr. Arnaldo, 455, São Paulo, SP CEP 01246-903 Brazil; 2 Department of Pathology, Universidade Do Oeste Paulista, Presidente Prudente, São Paulo Brazil; 3 Department of Physiotherapy, Universidade Federal Do Triângulo Mineiro, Uberaba, Minas Gerais Brazil; 4 Allergy and Immunology, Hospital das Clínicas, Federal University of Paraná, Curitiba, Brazil

## Correction to: Allergy Asthma Clin Immunol (2019) 15:50. https://doi.org/10.1186/s13223-019-0363-0

The original version of this article [1] unfortunately included an error to an author’s name: Author Raquel Annoni was erroneously presented as Raquel Anonni.

The author name has been updated in the author list of this Correction article.
